# Use of whole-cell bioreporters to assess bioavailability of contaminants in aquatic systems

**DOI:** 10.3389/fchem.2022.1018124

**Published:** 2022-09-30

**Authors:** Yi Zhu, Evrim Elcin, Mengyuan Jiang, Boling Li, Hailong Wang, Xiaokai Zhang, Zhenyu Wang

**Affiliations:** ^1^ School of Environmental and Civil Engineering, Institute of Environmental Processes and Pollution Control, Jiangnan University, Wuxi, China; ^2^ Department of Agricultural Biotechnology, Division of Enzyme and Microbial Biotechnology, Faculty of Agriculture, Aydın Adnan Menderes University, Aydın, Turkey; ^3^ School of Environmental Science and Engineering, Suzhou University of Science and Technology, Suzhou, China; ^4^ Biochar Engineering Technology Research Center of Guangdong Province, School of Environmental and Chemical Engineering, Foshan University, Foshan, China

**Keywords:** biosensor, environmental biotechnology, organic pollutant, heavy metal, environmental management, water pollution monitoring

## Abstract

Water contamination has become increasingly a critical global environmental issue that threatens human and ecosystems’ health. Monitoring and risk assessment of toxic pollutants in water bodies is essential to identifying water pollution treatment needs. Compared with the traditional monitoring approaches, environmental biosensing *via* whole-cell bioreporters (WCBs) has exhibited excellent capabilities for detecting bioavailability of multiple pollutants by providing a fast, simple, versatile and economical way for environmental risk assessment. The performance of WCBs is determined by its elements of construction, such as host strain, regulatory and reporter genes, as well as experimental conditions. Previously, numerous studies have focused on the design and construction of WCB rather than improving the detection process and commercialization of this technology. For investigators working in the environmental field, WCB can be used to detect pollutants is more important than how they are constructed. This work provides a review of the development of WCBs and a brief introduction to genetic construction strategies and aims to summarize key studies on the application of WCB technology in detection of water contaminants, including organic pollutants and heavy metals. In addition, the current status of commercialization of WCBs is highlighted.

## 1 Introduction

With the acceleration of industrialization, pollution of aquatic systems is increasing more rapidly than ever (Maslova et al., 2019; Liu et al., 2021), which is undoubtedly worse in areas where water resources are already scarce. It has been reported that over 420 billion cubic meters of sewage are discharged into freshwater systems and seas globally every year, resulting in the pollution of 5.5 trillion cubic meters of freshwater, which is more than 14% of the world’s total runoff (Liang and Yu, 2018). Due to water scarcity, sewage must be used to irrigate farmland in many places (Xue et al., 2019), which may reduce soil fertility and destroy soil structure, resulting in reduced crop yields (Urbano et al., 2017). In addition, food crops and vegetables tend to accumulate harmful substances such as heavy metals exceeding the allowable limit for pollutant intake, which threatens human health through the food chain (Lei et al., 2015). Therefore, the detection, monitoring and risk assessment of various pollutants in aquatic systems is an essential task for environmental decision makers and of critical importance for human development and ecological protection.

The concentration of pollutants is an important indicator for analyzing their environmental risks and their impact on ecology as well as human health (Alamgir et al., 2017; Majeed et al., 2021). In general, analytical methods used for the detection of contaminants in polluted water can be roughly divided into five categories: electrochemical, chromatographic, atomic spectroscopic, fluorescence-based, and colorimetric (Alhamlan et al., 2015; Zolkefli et al., 2020). The corresponding specific detection methods based on the principles of these five methods can be selected according to the target analyte(s). Atomic spectroscopy is usually used for the detection of heavy metals in aquatic systems; however, chromatography is the first choice when there is organic pollution (Petrovic et al., 2010; Rathi et al., 2021). The electrochemical method can be applied to the abovementioned situations simultaneously (Ding et al., 2021). Based on numerous studies in recent years on the detection of contaminants in polluted waters (Abdullah et al., 2018; Kalaiyarasan et al., 2020; Wang et al., 2020; Brahmkhatri et al., 2021), it can be seen that traditional methods are still preferred research tools since they remain as the workhorse for chemical identification and quantification. Chemical instrumental methods are used to analyze the total concentrations of pollutants (Kolpin et al., 2002). However, the total concentration of a contaminant is not always related to its toxicity, it has been demonstrated that bioavailability is a prerequisite for toxicity (Wells et al., 2005a; Fairbrother et al., 2007; Din et al., 2019). In other words, the chemical analysis data are inadequate to efficiently reflect the biological impacts of toxic substances (Axelrod et al., 2016). Therefore, the bioassays based on numerous biological entities including microorganisms such as microalgae, bacteria and yeasts are gaining importance in the last decades. Increasingly, during the environmental risk assessment process, the contaminants bioavailability has become the most important parameter relating to toxicity (Wells, 2012; Zhang et al., 2020a).

**FIGURE 1:**
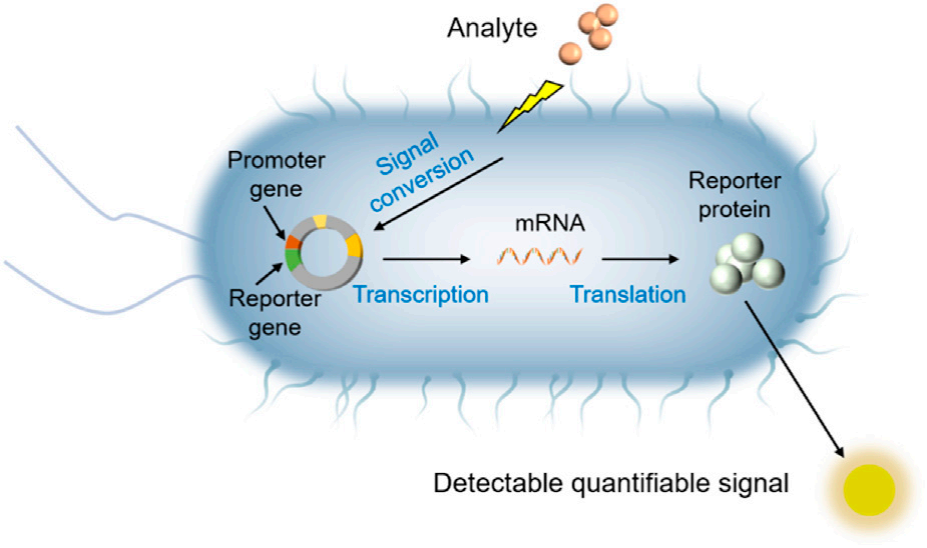
Diagram of the sensing mechanism of a bacterial Class I and Class II whole-cell bioreporters.

In recent decades, whole-cell bioreporter (WCB) technology has demonstrated excellent performance in pollution detection and offers extraordinary application potential (Elad and Belkin, 2017; Gui et al., 2017; Zhang et al., 2022a). WCBs are living organisms that are engineered to sense physical or chemical entities in the environment, and in response generate electrochemical or optical signals by the action of reporter genes as a “report” that can be easily quantified (See Figure 1, Belkin, 2003; Dhyani et al., 2022). Many studies have shown that WCBs can effectively detect various contaminants in aquatic systems, such as chromium (Cr) (Zhang et al., 2019), arsenic (As) (Sharma et al., 2013), lead (Pb) (Zhang et al., 2022a), pesticides (Tang et al., 2014) and petroleum hydrocarbons (Jiang et al., 2021). In addition, WCBs have been demonstrated to be simple, portable, cost effective, sensitive and rapid compared to traditional methods (Zhang et al., 2017; He et al., 2018; Zeng et al., 2021; Dhyani et al., 2022). That’s why WCB technology offers an ideal technique to address the shortcomings of analysis *via* chemical instrumental methods for environmental risk assessment. In this paper, we reviewed the current status of WCBs for the detection of aqueous contaminants. The development of WCBs and principles of their use are briefly presented. Then, recent studies on the detection of heavy metals and organic substances in aquatic environments using WCB technology is surveyed. Future research directions for WCBs are then discussed, which may offer guidance for environmental scientists who would like to consider working in this field.

## 2 The development of whole-cell bioreporter technology

A very early example of the development of WCB can be traced back to a seminal paper in 1990, when investigators in the laboratory of Gary Sayler constructed a bacterial sensor which can produce bioluminescence signal for naphthalene detection (King et al., 1990). Since then, WCB technology has been developing over more than 30 years, and genetically modified WCBs are being designed to detect the bioavailability or toxicity of an increasing number of environmental contaminants. WCBs can be divided into three categories: Class I, Class II and Class III, with classification based on how molecular recognition occurs, how recognition is converted into a signal and what parameter is sensed (van Der Meer et al., 2004). Class I and Class II are inducible WCB systems (lights-on) (Figure 1, Belkin, 2003; Wells et al., 2005b) emitting a measurable signal upon activation by target analytes and stress conditions, respectively, while Class III is a constitutive WCB system (lights-off) showing signal decrease upon exposure to toxic agents (Struss et al., 2010; Woutersen et al., 2011). Class I WCB is a target-specific strain that is constructed by using particular, tightly-regulated promoter involved in a specific genetic regulatory mechanism, thus can only recognize or detect the target chemicals (e.g., metals, hormones, antibiotics, organic solvents) and do so by expressing reporter protein in the cell causing an increase in the output signal (Harms et al., 2006). Class II WCBs are stress-specific WCB strains that can produce an increasing output signal upon exposure to cellular stress conditions (e.g., DNA damage, protein damage, oxidative stress, heat shock) (Belkin et al., 1996). For both Class I and Class II, a linear or monotonic increase in the signal produced by the reporter allows quantitative determination of the bioavailable concentration of the analyte (Boyanapalli, 2006; Harms, 2007). Class III WCBs are those with a normally high signal level that display a decrease in output signal upon toxic response to a wide range of chemicals or stress conditions (Muhammad et al., 2021). These WCBs are nonspecific since the decrease in metabolic activity could be derived from the cumulative effects of multiple contaminants in a sample. Compared with the Class II and Class III, Class I WCB is the most extensively studied category, mainly due to its ability to measure the bioavailability of individual analytes.

Despite decades of development, Class I WCB construction has kept the same design principle, which is essentially coupling a sensing element (regulatory promoter sequence) to a reporter element (source of signal) so that the reporter element can be controlled by the sensing element. This design involves an artificial fusion of the natural regulatory circuit in a bacterium with a promoterless gene coding for a detectable signal molecule, often a protein. When exposed to a bioavailable chemical species, a transcription regulator will activate the relevant promoter in the regulatory circuit, followed by expression of the reporter gene, thus producing quantitative output signal (Belkin, 2003; Zeng et al., 2021). A generalized conceptual illustration that provides a representative summary of the process is shown in Figure 1. Such WCBs have shown great potential for detecting the bioavailability of the contaminants of interest (Magrisso et al., 2008; Jiang et al., 2015; Brányiková et al., 2020). So far, most studies have focused on the construction strategies of WCB rather than improving the detection process despite significant developments in WCB technology and, while commercialized, this technology has not yet become commercially dominant compared to other approaches in environmental analysis. This is because developing useful WCBs is difficult for non-biological experts, and the only experts who make WCBs know how to use them well (Zhang et al., 2022a). Since WCB technology is based on a live cell, scientists also have been working on the immobilization of WCBs by integrating them into hydrogel polymers using different techniques such as entrapment, covalent attachment and encapsulation and employing these WCB-hydrogel on different platforms such as 3D-printed materials and fiber optics for their on-site monitoring application (Lopreside et al., 2019). However, how to use this technology remains unfamiliar to many scientific researchers and environmental practitioners. As mentioned above, WCB has unique advantages for measurement of contaminants in aqueous environments, thus, it needs to be a widely accepted technology.

The factors mentioned above have limited the dissemination of WCB technology and its commercialization. However, many studies have demonstrated successfully implementation of WCB in environmental research. In 2000, for the first time, Sayler and Ripp used the genetically modified microorganisms to monitor field bioremediation processes (Sayler and Ripp, 2000), demonstrating the field applicability of WCB technology. Since then, some microbial WCBs have been successfully commercialized. However, many of the commercially available WCBs belong to Class III, and it is crucial to develop more specific WCB assays (Class I) to be commercialized for the detection of specific contaminants.

## 3 Design and construction of whole-cell bioreporter

### 3.1 Regulatory genes

Regulatory proteins and reporter genes are an important part of WCBs, especially regulatory proteins have been referred to as the heart of the Class I and Class II WCBs (van Der Meer et al., 2004). In the classic method of constructing a WCB, the responsive genes plus promoter or only the promoter itself is fused to a reporter gene without its promoter. The fused gene elements can be constructed on a plasmid and then the recombinant plasmid can be transformed into a host cell (Gupta et al., 2019; Liu et al., 2022). When applied to the detection of target compounds or stress factors, the transcriptional regulators in these WCBs stimulate the promoters, which leads to the expression of downstream reporter genes and the increase of the output signal for Classes I and II (Shin, 2011). Therefore, the sensitivity and function of the WCBs depend on the responsiveness of regulatory gene circuits to the tested species.

The main mechanism of how regulatory proteins work involves an effector-binding response to chemical species with structures that enable cognate recognition (Bilal and Iqbal, 2019; Liu et al., 2022). In some cases, targets with structural similarity may all provoke a response. Studies have shown that regulatory proteins that react with benzene also react with ethylbenzene and xylene (Kim et al., 2005). For example, with respect to heavy metals, one study showed how the regulatory proteins that can detect cadmium (Cd) may also detect other heavy metal ions (Biran et al., 2000). In another example, Li et al. (2022) found that the construction of Pb-specific WCB can also be applied for the detection of Cd. Thus, WCBs can vary from highly specific to selective. Some investigators pursue specificity and selectivity studies to tailor selective *versus* specific response by random mutagenesis of promoters or amino acid substitutions of regulatory proteins. For example, Kim et al. (2020) modulated the selectivity of the promoter regulatory protein, ZntR, of *znt*-operon of *E. coli* which is used to sense Cd, mercury (Hg) and zinc (Zn). By site-directed engineering of the metal-binding region of ZntR protein, the WCBs in their study found to be able to sense new metalloid species such as Cr and Pb. In another study, Guo et al. (2019) showed that the sensitivity of the WCB can be enhanced by tuning the molecular ratio of the promoter regulatory proteins. Shpigel et al. (2021) aimed to enhance the performance of their WCBs for the detection of trace explosives, 2,4-dinitrotoluene (DNT) and 2,4,6-trinitrotoluene (TNT). They applied three rounds of random mutagenesis of promoters, *yqjF* and *azoR*, selecting the best performing variant in each round for the next one, and they reported that the WCB in their study had much lower background signal with a 3-fold reduction in the limit of detection for DNT. Over the decades, many studies have been conducted to gain insights into the molecular mechanisms for the construction of WCBs (Díaz and Prieto, 2000; Looger et al., 2003; Bilal and Iqbal, 2019) and these efforts have greatly promoted the development of increasingly diverse and fit-for-purpose WCB technology.

### 3.2 Reporter genes

Based on the output signal, widely employed reporter elements are genes encoding *β*-galactosidase (*lacZ*), luciferase (*luxCDABE*) and green fluorescent protein (*gfp*) (Su et al., 2011). Studies have found that *β*-galactosidase is relatively stable, and when X-gal (a split substrate of *β*-galactose bonds) is used as a substrate for hydrolysis, *β*-galactosidase catalyzes the production of blue products that are convenient for optical detection and observation (i.e., by eye or visible spectroscopy, Casadaban et al., 1983; Gierut et al., 2014; Wallenfels and Weil, 1972). Of course, when the reaction substrate is labeled with luminescence or fluorophore, the luminescence or excited fluorescence of the reaction can also be used as a detection basis (Xu et al., 2013; Doura et al., 2016). At the same time, if *p*-aminophenyl-β-D-galactopyranoside (PAPG) is used as a substrate, the p-aminophenol (PAP) produced by the enzymatic hydrolysis can also be detected by an electrochemical method, so as to determine the expression status of *β*-galactosidase (Matsui et al., 2006; Biran et al., 2010). Owing to these advantages, the *lacZ* gene is widely used as a reporter gene in genetic engineering. In recent years, researchers combined *lacZ* gene with the gene encoding urease for the construction of WCB to detect Cd and showed a very low limit of detection of 10 ppm (Shetty et al., 2003). In addition, the *lacZ* gene can also be used to construct WCB for the detection of organic pollutants. Peng et al. (2010) constructed *Escherichia coli* bioreporter using the *lacZ* gene for detection of phenol, they found that the *β*-galactosidase activity of PEDRP34 was about twice that of PEDRP14 with 0.1–5 μM, also implicating the importance of the host strain (*vide infra*).

**FIGURE 2:**
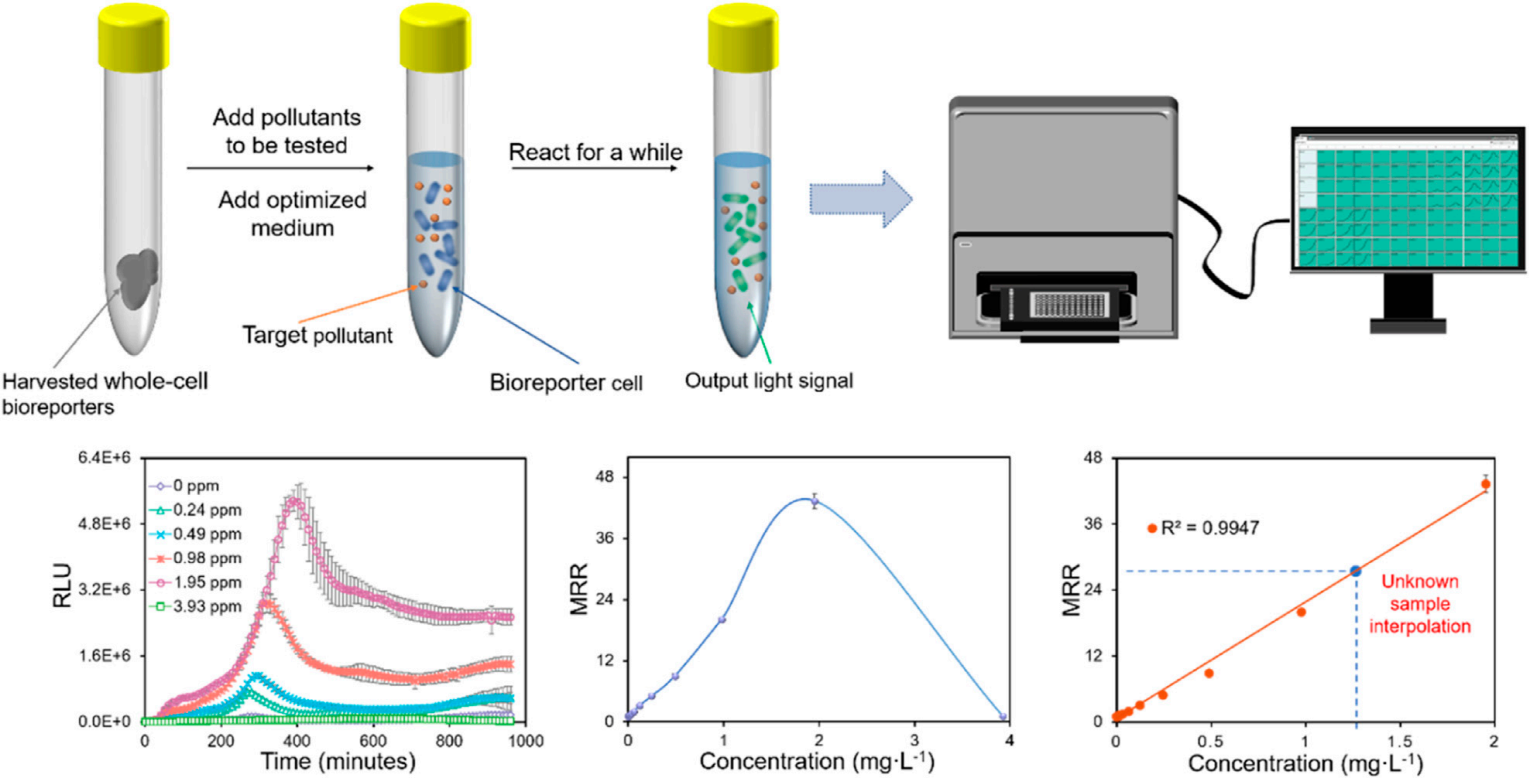
In the specific experimental process, the bioluminescence signal intensity is analyzed by fitting the curve under different concentrations or time periods of the pollutants to be tested (taking Zn as an example, unpublished data).

Bioluminescence, which uses luciferase to produce light is another most commonly used reporter protein for the design of WCBs. Engebrecht et al. (1983) isolated the complete *lux* cassette (*luxCDABE*) from bioluminescent bacteria, *Vibrio fischeri*, and the recombinant *E. coli* constructed on this basis was the first to generate bioluminescence without aldehyde addition. Luciferase can be found in various organisms such as firefly luciferase (encoded by *luc*) and sea pansy luciferase (encoded by *ruc*) (Wilson and Hastings, 1998), and bacterial luciferase-based reporter elements (*luxAB* or *luxCDABE*) are employed frequently by WCBs (Close et al., 2009). It has long been shown to be applicable in research such as the usage of WCBs for detection of contaminants and even the number of bacterial species (Ilina et al., 2018). Under aerobic conditions, bacterial luciferase oxidizes reduced riboflavin mononucleotide (FMNH_2_) and long-chain aldehydes, and this reaction produces a blue-green light with a peak wavelength of 490 nm (Close et al., 2012). Bhattacharyya et al. (2005) constructed five luminescent WCBs for the measurement of chlorinates in groundwater and found that the WCB results agreed well with the chemical analyses. Figure 2 shows a specific experimental process of how bioluminescence-based WCB works from our previous studies; however, in practice the same process may be applicable to other reporter genes that produce an optical signal.

The green fluorescent protein (GFP) firstly isolated from crystal jellyfish *Aequorea victoria* (Ormö et al., 1996) is also an important reporter element. Its potential to be successfully expressed in a wide variety of species without any exogenous substrate or ATP requirements along with its small size (238 amino acids) are the reasons why it has become a commonly used reporter protein (Oertner, 2006). GFP is an auto fluorescent protein emitting a fluorescent signal at 508 nm upon ultraviolet or blue light excitation, and requires only oxygen for its expression (Craggs 2009). The shortcomings of the *gfp*-based WCBs are a high background signal and a slow response to contaminants (Shin, 2011). Many studies have shown that the enhanced green fluorescent protein (EGFP) has superior stability, emit brighter fluorescence and *egfp*-based WCBs have become an effective tool for detection of bioavailable species (Roberto et al., 2002; Wells et al., 2005c).

In addition to the aforementioned commonly used reporter genes for the construction of bioreporters, some other genes can also be incorporated into the design and construction of WCBs. For example, the *Renilla* luciferase gene, which only requires coelenterazine and oxygen to produce blue light with a wavelength of 480 nm after expression (Farzannia et al., 2015). Another interesting gene is *crtA* which is responsible for carotenoid synthesis (Yagi, 2007), has also been used as reporter gene for the construction of WCB.

### 3.3 Host strain

When constructing a WCB, besides the reporter gene, the choice of host strain is also particularly important. A WCB is a living organism that is typically derived from bacteria as a host strain, but fungi, yeasts or microalgae can also be used. And the cell functional strategies are also different when exposure to pollutant which is mainly governed by regulatory genes and reporter genes. For different target compounds, suitable organisms with relevant detoxification, degradation or other metabolic pathways for a particular contaminant or group of contaminants should be selected as host cells also considering the actual environmental conditions. This is because the sensitivity and response of individual strains vary under the stress of different contaminants (Phyo et al., 2021). For example, for the construction of WCB to detect mercury, Din et al. (2019) isolated mercury-resistant bacteria, an *Enterobacter cloacae* strain, from a contaminated soil environment. They reported that their bioreporter was both more sensitive and faster than other bireporters constructed with model *E. coli* bacterial strains. Han et al. (2019) isolated salt-tolerant *Platymonas subcordiformis* from sea species of motile microalgae for the construction of WCB and found that this WCB showed good sensitivity to Cu under a wide range of salinity, temperature and light levels. In addition, the host cell can also set some restrictions during the detection process, such as induction temperature and test media used to culture the WCB (Hynninen and Virta, 2010). However, some strains for constructing WCBs are isolated from wild-type bacteria that can survive in a very wide array of mineral media.

The bacterium *E. coli* has been widely used as a host cell for WCB to measure the genotoxicity of typical environmental contaminants such as inorganic and organic compounds (Yoon et al., 2016; Ali et al., 2021). However, in addition to *E. coli*, other Gram-negative bacteria such as *Alcaligenes eutrophus* (Peitzsch et al., 1998; Leth et al., 2002) and *Cupriavidus metallidurans* (Magrisso et al., 2009) as well as Gram-positive bacteria such as *Bacillus subtilis* and *Staphylococcus aureus* (Tauriainen et al., 1998; Bondarenko et al., 2008) have also been widely used for the construction of WCBs. Aside from bacteria, fungi, yeasts and algae (Sanseverino et al., 2009; Bullerjahn et al., 2010) have been used as hosts for contaminant detection, albeit to a lesser degree.

## 4 Application of whole-cell bioreporter for the measurement of pollutants in aquatic environment

As discussed above, compared with the traditional methods of contaminant detection, WCB technology reflects the bioavailability of contaminants and is capable of being more sensitive, time and labor saving. In recent years, many studies have been conducted using this technology to measure different types of bioavailable contaminants in aquatic systems. However, as mentioned earlier, the popularization of this technology to environmental researchers is the key to promoting its development into a standard detection method for environmental pollutants. In this section, some recent studies that primarily focus on Class I WCBs were reviewed, at the same time, Class II and III WCBs were briefly discussed.

### 4.1 Organic contaminants

Organic pollutants may enter aquatic systems through different ways, for example, direct discharge of untreated industrial or domestic sewage, oil spills from shipping or offshore exploration, and infiltration of soil contaminants such as pesticide spraying (Kostianoy et al., 2020; Lamba et al., 2020; Zhou et al., 2020). Recent studies have reported WCBs as detection tools for measuring bioavailable organic pollutants in water and achieved considerable results (Jiang et al., 2019; Muurinen et al., 2019; Voon et al., 2022). The first WCB was constructed for the detection of naphthalene which is an organic pollutant (King et al., 1990). Since then, many kinds of organic pollutants such as phenol (Han et al., 2019), amoxicillin (Zhao et al., 2021), chloroform (Ma et al., 2020), naphthalene (Sun et al., 2017), and testosterone (Moscovici et al., 2020) have been evaluated. Table 1 summarizes the recent studies on the application of WCB to detect organic compounds in water. As shown in Table 1, the sensitivities of WCBs toward many organic contaminants, such as naphthalene, phenanthrene and toluene, have been achieved in μg/L range similar to the sensitivity of instrumental analysis (He et al., 2018; Hurtado-Gallego et al., 2018; Muurinen et al., 2019). This indicates the WCB technology is sensitive enough to assess the environmental risk of organic contaminants and approach the allowed concentration by environmental regulations in the mg/L‒level (WHO, 2021, in which the allowable concentrations of organic contaminants in drinking water are listed).

**TABLE 1:** Whole-cell bioreporters used in the detection of organic pollutants.

Analyte(s)	Host strain	Detection limit	Reporter gene	Comments	References
Crude oil	*E. coli*	—	*luxCDABE*	The sensitive response of the WCB indicates that over time, the genotoxicity of the seawater contaminated by crude oil is reduced	Jiang et al. (2017)
Toluene	*E. coli*	4.6 μg/L	*luxCDABE*	The developed WCB can detect toluene in actual water bodies and the detection performance is very well correlated with the GC-MS analysis	Wang et al. (2021)
	*Pseudomonas putida*	—	*luxCDABE*	Freeze-dried strains have low luminescence activity to toluene	Ko and Kong, (2017)
Three xylene isomers	*Pseudomonas putida*	—	*luxCDABE*	WCB has the potential to initially detect the contamination of groundwater by gasoline leaks	Ko and Kong, (2017)
Methyl benzyl alcohol		—			
Naphthalene	*E. coli*	1.28 μg/L	*luxCDABE*	It has significant advantages in fast *in situ* measurement	Sun et al. (2017)
2,3,7,8-tetrachlorodibenzo-p-dioxin (TCDD)	*Saccharomyces cerevisiae*	161 ng/L	*luxCDABE*	The WCB can detect the presence of TCDD in artificially contaminated fish samples	Xu et al. (2018)
β-naphthoflavone		272.3 ng/L			
Diclofenac	*S. cerevisiae*	2.96 mg/L	*tgfp* (turbo green fluorescence gene)	Engineered yeast cells embedded in a microfluidic device enabled detection of nonsteroidal anti-inflammatory drug in synthetic wastewater	Schirmer et al. (2018)
Triclosan	*Nostoc*	0.498 μg/L	*luxCDABE*	The WCB is highly specific in detecting oxidative stress products in fresh water	Hurtado-Gallego et al. (2018)
Simazine, atrazine, diuron	*Chlorella vulgaris* and *Tetrahymena pyriformis*	—	‒	The strain responded to the presence of multiple herbicides by means fluorescence decrease	Turemis et al. (2018)
17β-estradiol	*S. cerevisiae*	0.032 μg/L	*egfp*, *mRuby2, mTagBFP2*	Yeast-based WCBs have the potential to be applied to the detection of endocrine disruptors in wastewater	Moscovici et al. (2020)
Testosterone		0.070 μg/L			
Mono aromatic hydrocarbon	*E. coli* DH5α	‒	*gfp* and *cfp*	Both strains are highly sensitive to aromatic hydrocarbons	Patel et al. (2019)
Polyaromatic hydrocarbons	*E. coli* DH5α				
Tetracycline	*E. coli*	5 μg/L	*luxCDABE*	The use of polyester swabs together with luminescent WCB can be used for the detection of contaminants under field conditions	Muurinen et al. (2019)
Carbendazim, profenofos, cypermethrin, carbaryl, chlorpyrifos	*E. coli*	—	*lacZ*	WCBs can detect the genotoxicity of several pesticides and at the maximum residue limits permitted in agriculture commodity of pesticide mixtures thereof	Chumjai and Vangnai, (2020)
Ethanol	*E. coli*	1% (v/v)	*luxCDABE*	Bacteria immobilization technology is combined with smart phones to facilitate real-time monitoring of water environmental toxicity	Ma et al. (2020)
Chloroform	*P. aeruginosa* PAO1	0.02% (v/v)			
Sodium Dodecyl Sulfate (SDS)		0.1 mg/L	*gfp*	The first WCB for specific detection of SDS. The biosensor has high sensitivity and low limit of detection	Dey et al. (2020)
Amoxicillin	*Acinetobacter baylyi*	—	*luxCDABE*	This method detects the bioavailability of the process of degrading amoxicillin	Zhao et al. (2021)
Formaldehyde	*E. coli* MG1655	1.5 mg/L	*gfp*	Whole-cell genotoxicity WCB can detect formaldehyde in waters in drinking water permissible limits	Elcin et al. (2021)
Allylthiourea	*E. coli* DH5α	1 μg/L	*gfp*	The WCBs developed could detect nitrification inhibitor allylthiourea in real wastewater samples and can be used for nitrification process status	Zappi et al. (2021)

In the aqueous environments, many different types of contaminants often coexist. Studies have also shown that one type of WCB can be used for detection of multiple different organic pollutants (Ko and Kong, 2017; Patel et al., 2019) which is an innovative technology for target contaminants. Patel et al. (2019) found that the WCB used in their study was highly sensitive to both monoaromatic and polyaromatic hydrocarbons in industrial wastewater samples, with a detection limit in the range of 0.1–1 μM. Chumjai and Vangnai (2020) also reported that WCB can measure the genotoxicity of a variety of combined pollutants. The hidden mechanism could be that regulatory proteins can respond to chemicals with similar structure that can fit into effector-binding, as discussed in Section 3.1. In addition, some WCBs have been constructed for assessment of one specific contaminant bioavailability and have achieved good results. For instance, Sun et al. (2017) showed that the WCB adopted by the study can effectively respond to a low concentration of 0.01 μM naphthalene, showing high specificity and sensitivity and not responding to other polycyclic aromatic hydrocarbons. Wang et al. (2021) also demonstrated that the WCB they used had a low limit of detection and a wide measurement range (from 0.05 to 500 μM) for the detection of the specific pollutant, toluene.

Antibiotic residues in water can strongly affect water quality which may also arouse the accumulation of antibiotic resistance genes. In order to construct WCBs for detection of an antibiotic, an antibiotic-sensitive promoter should be fused with a reporter gene (Parthasarathy et al., 2018; Ma et al., 2020). Melamed et al. (2012) investigated the response of different WCBs and found that each WCB can respond to a specific antibiotic. They thus demonstrated the ability of WCBs for the detection of unknown antibiotics in natural water. Recently, a tetracycline WCB was constructed for measuring water samples that had a detection limit of 5 μg/L making measurements in field conditions possible (Muurinen et al., 2019). Under natural conditions, differences in physical and chemical properties of water will be an important challenge factor affecting the performance of WCB. Zhang et al. (2014) found that pH strongly affects the measurement of bioavailable tetracycline. Therefore, it is of great importance to construct WCBs with high activity in complex water environments.

### 4.2 Heavy metals

Compared with organic pollutants, heavy metals have more adverse effects on global public health and various types of ecosystems due to their toxicity, mobility and persistence (Pujari and Kapoor, 2021). To cope with the complex requirements from environmental elements and necessary technical support, the WCB is a good tool for detection of heavy metal contamination.

The assessment of the heavy metal bioavailability is a hot topic in the research of WCBs. Table 2 summarizes the studies that applied WCB technology to detect various heavy metals such as (van Genuchten et al., 2018), Cr (Kim et al., 2020), Cd (Hui et al., 2021), copper (Cu) (Pang et al., 2020), mercury (Hg) (He et al., 2018), antimony (Sb) (Kim et al., 2020), Pb (Zhang et al., 2017), silver (Ag) (Hurtado-Gallego et al., 2019a), iron (Fe) (Blanco-Ameijeiras et al., 2019), titanium (Ti) (Hurtado-Gallego et al., 2019a), zinc (Zn) (Kim et al., 2020), cobalt (Co.) (Martin-Betancor et al., 2017), and nickel (Ni) (Cayron et al., 2017). Different WCBs may have different sensitivities for the same metal. A WCB *Acinetobacter* DF4/PUTK2 strain carrying luciferase genes *luxCDABE* was constructed for the measurement of Pb in water by Muhammad et al. (2021), who found that the WCB is sensitive to Pb in the concentration range from 19.25 to 15,000 mg/L. Kessler et al. (2012) demonstrated that *E. coli zntA* promoter could effectively detect Pb in a concentration range from 0.0012 to 12.5 mg/L and that a toxic effect appeared at higher Pb concentrations (>12.5 mg/L). Therefore, different WCBs can be selected according to the level of Pb pollution in targeted water. For example, the WCB *Acinetobacter* DF4/PUTK2 may be a good tool for detection of higher Pb concentrations in wastewater, whereas *E. coli zntA* could be applied to detect the lower Pb concentrations in drinking water (Kessler et al., 2012; Muhammad et al., 2021).

**TABLE 2:** Whole-cell bioreporter used for heavy metal detection in aquatic system.

Analyte(s)	Host strain	Detection limit	Reporter gene	Comments	Reference
Ag, Zn, Co.	*Synechococcus elongatus*	—	*luxCDABE*	Among the factors that constitute the bioavailability of metals, free ionic metals are the main component	Martin-Betancor et al. (2017)
As (Ⅲ)	*Synechocystis* PCC6803 (Cyanobacteria	0.3 mg/L	*luxAB*	The promoter of the *arsB* gene encoding an arsenite-specific transporter can be used in the construction of WCBs	Peca et al. (2017)
Sb (Ⅲ)		0.49 mg/L			
As (Ⅴ)		11.24 mg/L			
Hg	*E. coli*	200.59 μg/L	*PpFbFP* (flavin-based fluorescence gene)	Under marine environmental conditions, the bioavailability of Hg is related to ionic strength	Stenzler et al. (2017)
As	*E. coli*	7.1 μg/L	*egfp*	The detection limit is lower than the WHO drinking water requirement and can be applied in practice	Zhang et al. (2017b)
Hg	*A. baylyi ADP1*	20 μg/L	*luxCDABE*	This type of WCB can be used to detect the toxicity of heavy metals in marine environments	Cui et al. (2018)
Zn		1 mg/L			
Cu		0.1 mg/L			
Cd		50 μg/L			
Cd	*E. coli* BL21 (DE3)	0.28 mg/L	*egfp*	The WCB is only sensitive to Cd and Hg	Kang et al. (2018)
Hg	*E. coli* BL21	0.3 mg/L	*luc* (firefly luciferase gene)	The designed WCB is sensitive to Cu	Taghavi et al. (2018)
Cu		6.35 mg/L			
Zn		5.88 mg/L			
Fe		5.58 mg/L			
As (Ⅴ)	*E. coli* DH5α	5 μg/L	*luxCDABE*	Flocculation particle size affects the As bioavailability attached to iron-based oxides	van Genuchten et al. (2018)
As (III)	*E. coli* DH5α	—	*luc*	Research on non-uniform base pairs contributes to the optimization of WCBs	Chen et al. (2019)
Hg	*Enterobacter cloacae*	0.2 μg/L	*luxABCDE*	The bacteria may become an alternative to commonly used host cells	Din et al. (2019)
Pb		0.39 mg/L			
Cu	*P. fluorescens*	—	*luxAB*	This method overcomes the interference of pH when measuring Cu	Hansen et al. (2019)
Ag	*Synechococcus elongatus* and *Nostoc*	120 μg/L	*luxCDABE*	WCBs can be used to detect the toxicity of metal nanoparticles	Hurtado-Gallego et al. (2019a)
Cu		1.6 mg/L			
Ti		15 mg/L			
Zn		60 μg/L			
Cr (Ⅴ)	*Acinetobacter baylyi*	—	*luxCDABE*	WCBs are used to indicate changes in biological toxicity during Cr bioremediation	Zhang et al. (2019)
Cr (Ⅲ)					
Cd	*E. coli* MG1655	2 μg/L	*gfp*	The WCB has strong specificity to Cd, and the detection limit is just suitable for drinking water requirements	Elcin and Öktem, (2020)
As, Sb, Cd, Cr, Ni, Hg, Pb, Zn, Cu, Au	*E. coli* DH5α	—	*egfp*	The metal-sensing properties and selectivities of the WCB can be modified through changing amino acid sequences of promoter regions	Kim et al. (2020)
Cu	*E. coli*	15.7 μg/L	*gfpmut2*	Through genetic improvement, the environmental stability and adaptability of the WCB are improved	Pang et al. (2020)
Pb	*E. coli*	1.2 μg/L	*luxCDABE*	Evaluate and model the bioavailability and corresponding existing forms of Pb in Lake Tai	Zhang et al. (2020b)
Cd (Ⅱ)	*E. coli* TOP10	5.6 μg/L	*egfp, mcherry*	Two sets of sensor systems are used to detect the bioavailability of Cd in natural water environments	Hui et al. (2021)
As	*E. coli* TOP10	0.75 μg/L	*gfp*	Designed and constructed a tool that can be practically used to detect the bioavailability of As in water samples	Li et al. (2021)
Hg (Ⅱ)	*E. coli* TOP10	0.58 ± 0.07 μg/L	*luc*	Adopt a variety of sensing mechanisms to simplify the analysis and operation process, and it is easy to detect sewage on the spot	Lopreside et al. (2021)
Pb	*Acinetobacter*	19.25 mg/L	*luxCDABE*	The WCB DF4/PUTK2 is a good tool Pb detection in water with a wide detection concentration range	Muhammad et al. (2021)
Hg, As, Pb, Cd, Zn, Cu	*E. coli* MG1655	—	*gfpmut2*	WCBs can be used to screen out metal mixed contaminants that need to be detected by traditional methods	Cregut et al. (2022)

When constructing WCBs, it is important to note possible toxic effects of analytes. It has been reported that heavy metals (particularly non-essential metals) can be toxic to most bacteria at exceptionally low concentrations (Lemire et al., 2013). Therefore, in many WCBs, the detoxification genes can be selected to enhance the tolerance of WCB to heavy metals (Zeng et al., 2021). For instance, *zntA* and *cadA* which are two typical metal transporter protein-coding genes can be used to engineer WCBs to survive in extreme environments (Biran et al., 2000). Zhang et al. (2022b) demonstrated that *zntA*-based WCB can measure Pb bioavailability in a wide concentration range of 1.2 μg/L–12.5 mg/L, which makes this *zntA*-based WCB an effective tool for environmental Pb pollution risk assessment. Deletion of metal exporter genes (such as *copA*) can cause intracellular heavy metal accumulation, which may enhance the sensitivity of WCBs at low metal exposure conditions (Kang et al., 2018). Therefore, the difference in detection limits of WCBs is the key to determine at which level of water pollution detection they can be applied to. As shown in Table 2, the limit of detections of many WCBs for Cd, Hg, Cu and As in water are at the μg/L level, which are comparable to allowable limits for metals in water set in National Primary Drinking Water Regulations (US EPA, 2017). This further indicates that WCB technology can be applied for accurate environmental risk assessment.

Heavy metals can occur in different forms which can strongly affect the metal bioavailability and also determine the dose response of WCBs during the detection process. Zhang et al. (2017) used WCB for the detection of Pb and found that Pb can combine with the ligands such as EDTA, humic acid and natural dissolved organic carbon in aqueous solution which makes it less bioavailable. They also showed that the bioreporter data agreed well with the speciation model result. Another related study showed that when considering the effect of dissolved organic carbon as a ligand on the effect of Pb bioavailability, the WCB data were also in good agreement with the speciation model data (Zhang et al., 2020a). Since the speciation model has been widely used in the environmental risk assessment of heavy metal pollution, this study further confirmed the possibility of applying the WCB technology to the risk assessment of Pb in natural waters. Moreover, slight differences in experimental conditions such as pH, cell density and medium composition may result in different detection limits (Zhang et al., 2022a). Of course, selecting the most suitable carrier strain is also an important measure to reduce the detection limit of the target heavy metal (Ivask et al., 2009). Both *Acinetobacter baylyi* (Cui et al., 2018) and *Platymonas subcordiformis* (Han et al., 2019) were used as host cells for the constructing WCBs and testing bioavailable Cu in water. The difference between the two experimental results is that the detection limit of the latter is much lower than that of the former. The possible reason could be that Han et al. (2019) selected microalgae in the selected sea area, which are salt-tolerant and best adapted to temperature and radiation. Compared with the bioreporter strain of Cui et al. (2018), it had a higher fitness for the measured environment. The relationship between the concentration of metal cation and its bioavailability is evident; it is worth noting that both nitrate (NO_3_^-^) and carbonate (CO_3_^2-^) ions can modulate the bioavailability of the metal (Rodea-Palomares et al., 2009). This demonstrates that the chemical properties of water environment are also the key factors affecting the detection results of WCB when used to detect the bioavailability of heavy metals in natural water. To our knowledge, there is no standard WCB method for environmental risk assessment. To address this issue, standard validation methods that taking environmental factors into consideration need to be combined with WCB research to facilitate their application to environmental risk assessment.

### 4.3 Class II and class III bioreporters

In addition to the widely investigated Class I WCB, other types of WCBs, namely Class II and Class III are also commonly used. In fact, these two types of WCBs have been more widely commercialized for water monitoring. Although the target analyte varies in different studies, the key point is that the stress response mechanism of Class II WCBs is under external pressure. Numerous studies have been carried out for investigation of the oxidant and antioxidant properties of chemicals by using WCBs (Senevirathna et al., 2009; Lim et al., 2019). To investigate the oxidative stress of the pollutants, as early as 1996 in Belkin’s laboratory, *E. coli* DPD2515 and *E. coli* DPD2511 strains of WCBs were constructed for the detection of hydrogen peroxide (H_2_O_2_) and superoxide (O_2_^−^). They further indicated that the WCBs can be applied for environmental monitoring and studying cellular responses to oxidative hazards (Belkin et al., 1996). Hartono et al. (2018) explored the oxidative stress and cellular damage of multiwalled carbon nanotubes (MWCNT) to WCB and observed that membrane damage is a major cause of MWCNT toxicity. Hurtado-Gallego et al. (2019b) used cyanobacterial bioreporters for (reactive oxygen species) ROS detection and found that both WCBs can detect oxidative stress caused by emerging pollutants in aquatic system.

Class III WCB is also known as ‘lights-off’ WCB, which is an extension of the biotoxicity method (Belkin, 2003). The mechanism of this WCB is that a decrease in light emission is detected upon exposure to toxic compound(s) and/or stress conditions. This is, in whole or in part, because the ATP or reducing power required by cellular metabolism for the generation of signal (e.g., bioluminescence) may be affected by any factor that has an influence on this metabolism, thus resulting in a decrease of signal output (Mateo et al., 2015). Studies have shown that cyanobacterial bioreporters constructed for Fe detection in water, light emission decreased when increasing concentrations of Fe are supplied to the WCBs (McKay et al., 2005; Hassler et al., 2009). The strain of *Synechococcus* sp. PCC7942 was constructed as a WCB by fusing *phoA* promoter to reporter genes *luxAB* from *Vibrio harveyi* to detect phosphorus (P) (Gillor et al., 2002). This *phoA* gene promoter is induced in P-deficient environment, and as the P concentration increases, luminescence decreases (Gillor et al., 2002). In addition to detecting the nutrient bioavailability, Class III WCBs can also be applied to detect general toxicity. Abd-El-Haleem et al. (2006) showed that the bioluminescence lights-off took place when the WCB was used for the detection of different heavy metals (Cu, Fe, Ni, Zn, Co., Cd, and Cr). Similarly, Muhammad et al. (2021) demonstrated that “lights-off” WCB was effective for Pb detection.

## 5 Outlook and perspectives

The WCBs described in this review have been demonstrated to be an ideal tool for the detection of contaminants in aquatic systems. Even though this technology has numerous advantages, several considerations must be addressed before WCBs can be more widely accepted and commercialized, including further shortening the response times, increasing cell sensitivity and improving cell selectivity. In addition, how to construct a useful WCB that can exhibit good performance in detection of contaminants is of high importance. Moreover, during the detection processes, the chemical components of the culture medium, pH of the medium and cell concentration at harvesting are important parameters that may strongly affect the performance of WCBs. Therefore, it is essential to provide guidance to both non-bioengineer researchers and environmental practitioners, and efforts should be made toward the development of standard methods. While this has been realized to some extent for certain types of WCBs, most prominently Class III WCBs, such guidance and standard methodology is not yet mature for Class I WCBs. The dissemination of a greater array of commercially available Class I WCBs would be very helpful in this regard, which will also promote their application in the field of environmental risk assessment.
